# Phase Angle as a Predictor for Physical Function in Institutionalized Independent Older Adults

**DOI:** 10.3390/ijerph192114615

**Published:** 2022-11-07

**Authors:** Ana Morais, Soraia Ferreira, Alexandre Duarte Martins, Pablo Tomas-Carus, José Marmeleira, Jose A. Parraca

**Affiliations:** 1 Departamento de Desporto e Saúde, Escola de Saúde e Desenvolvimento Humano, Universidade de Évora, 7004-516 Évora, Portugal; 2 Comprehensive Health Research Centre (CHRC), University of Évora, 7004-516 Évora, Portugal; 3 Life Quality Research Centre, 2040-413 Rio Maior, Portugal

**Keywords:** bioelectrical impedance, functional fitness, elderly, body composition

## Abstract

The aim of this study was to investigate the relationship between phase angle (PhA) and physical function in institutionalized, independent older adults. Physical function was evaluated using the Senior Fitness Test Battery. PhA was measured by electrical bioimpedance at 50 khz, and body composition parameters were also registered. Results showed that PhA significantly correlated with all physical fitness tests, except for arm curls. Regarding the results of the multivariate analysis, three models were created: Model 1, formed by a dependent variable “PhA” and two predictor variables “8 ft up-and-go” and “6 min walk”; Model 2, formed by a dependent variable “PhA” and three predictor variables “8 ft up-and-go”, “6 min walk” and “30-s chair stand”; and Model 3, formed by a dependent variable “PhA” and four predictor variables “8 ft up-and-go”, “6 min walk”, “30-s chair stand” and “arm curl”. Results showed that predictor variables had a significant influence on the PhA for all three models (Model 1: *p* = 0.001, 12.5%; Model 2: *p* = 0.002, 12.9%; and Model 3: *p* = 0.005, 13.1%). For women, Model 1 showed a significant influence of predictor variables on the PhA (*p* = 0.030, 9.3%). The results for men in Models 1, 2 and 3 showed significant influences on the PhA (*p* = 0.002, 31.2%; *p* = 0.006, 31.6%; and *p* = 0.016, 31.6%; respectively). This study confirmed previous studies regarding to the relationship between PhA and physical function. It also indicates that PhA could be an excellent predictor of physical function.

## 1. Introduction

In recent years, aging has been a crucial topic for scientific research, namely focused on cognitive and physical factors or skills [1]. Significant concepts, such as “healthy aging” or “longevity” are accepted and promoted by society [2]; however, it is important to objectify the parameters that should be the center of programs and interventions for older adults. It has been suggested that physical function in older adults is influenced by a loss of muscle mass, an increase of fat mass, high levels of interleukin (IL)-6 and C-reactive protein, loss of walking speed [3,4,5,6], decrease in the sensorimotor function and changes in the nervous system [7] that reduce muscle strength, power, balance and functional capacity performance [1,8,9]. From a clinical and preventive perspective, the main focus is to reduce the possible onset of chronic diseases such as diabetes, cancer or cardiovascular conditions. Body composition assessment allows clinicians to obtain information about health, including nutritional and physical function, in older adults [1]. Specifically, phase angle (PhA) is considered a clinically important parameter that provides information about the health and integrity of cells [10], and is being increasingly applied to the elderly. However, many studies have focused on children due to their close relationship with maturation stages [11]. Additionally, PhA seems to constitute an important indicator in physical assessment and the elaboration of guidelines for physical activity and exercise, as well as health-related physical fitness [12]. On the other hand, physical activity has a positive effect on PhA in healthy and non-healthy individuals [13]. As previously explained [1], PhA is obtained by bioelectrical impedance analysis (BIA). BIA is a body composition assessment method that is non-invasive and easy-to-use. It measures the opposition of body tissues to a low-level flow, changing the radiofrequency of an electric current [14]. The PhA is calculated using two parameters: bioelectrical resistance (R) and reactance (Xc). It has been used in research in all age groups and findings have suggested that PhA has a growing trend, from childhood until adulthood, in healthy individuals [14] and is higher in males [15]. Generally, a high PhA is associated with higher cellularity and better cell membrane or cell function [14]. However, research focused on children has concluded that body composition parameters should be taken into account while investigating PhA and fitness variables [11]. The findings of different studies have suggested a strong relationship between PhA and muscle strength [12,16]. Direct association between aerobic fitness and PhA is mostly found in research with children and adolescents, with a lack of studies involving adults and older adults [16]. However, in adults, recent findings have suggested that PhA could constitute a predictor of conditions, such as sarcopenia [1,17] or nutritional risk [18]. In addition, research conducted with obese adults found a direct association between PhA and the maximum strength of the upper and lower limbs [19]. The same study also found an inverse association between PhA and body fat levels. Studies conducted with patients with chronic diseases indicated that PhA levels were associated with lower risk of morbidity and mortality, and were especially related to cardiovascular events [13]. Additionally, research conducted with breast cancer survivors found significant correlations between PhA values and muscular strength and muscular endurance levels [20]. These findings strengthen the idea that physical activity could be considered a treatment for these patients.

Therefore, this study aimed to investigate the relationship between PhA and physical function and to verify the ability of PhA to predict the physical function in institutionalized, independent older adults. We hypothesized that PhA is associated, and can predict physical function in this population.

## 2. Materials and Methods

### 2.1. Study Design and Participants

A total of 111 volunteers were accepted to participate in this cross-sectional study. The participants were independent and were recruited from seven nursing home residences in the region of Évora (Portugal); the inclusion criteria were: living or using a daycare center in a nursing home residence, age of 65 years or more and having autonomy in locomotion, as well as being able to use mobility support. Exclusion criteria were defined as: being in a wheelchair, needing help from a team member to get around, and not understanding the guidelines provided by the evaluator.

The volunteers were informed about the study and its goals, and signed an informed consent form regarding the study. The University of Évora ethics committee approved the study (cod: GD/21849/2017) in accordance with the Declaration of Helsinki [21].

### 2.2. Procedures

Body composition and physical function data were collected by a kinesiologist. The participant’s height in meters (m) was measured without shoes according to ISAK standards. The weight in kilograms (kg) and PhA in degrees (°) were measured by electrical bioimpedance using a Tanita MC-780. Body mass index (BMI) was calculated from weight divided by the square of height. Waist circumference in centimeters (cm) was measured with a tape at the level of the uppermost lateral border of the iliac crest.

Physical function was evaluated using the Senior Fitness Test Battery [22]. The strength of upper and lower limbs was evaluated using a 30-s chair stand (rep) and arm curl (rep), respectively. The flexibility of upper and lower limbs was evaluated using a chair sit-and-reach test (cm) and a back scratch (cm). The 8-ft up-and-go test (sec) was used for assessing agility and dynamic balance, and the 6-min walk evaluated aerobic endurance. In the 30-s chair stand, the participant is sitting on a chair with arms folded across the chest and has to stand up and sit down as often as possible for 30 s. The arm curl is performed with the participant seated, and the goal is to do as many bicep curls as possible in 30 s. This test is accomplished with the dominant arm, and the hand weight for men is 8 lb and for women it is 5 lb. The score in these two tests is the maximum number of repetitions. In the sit-and-reach chair test, the participant is sitting in a chair as far forward as possible with one leg extended. The participant places one hand over the other and tries to reach as far forward as possible without bending the knee. For the back scratch test, the participant is standing and has to put one hand over the shoulder and the other in the middle of the back. The goal of this test is to try to bring or overlap the hands together. The score for the chair sit-and-reach test and back scratch is the mean of three repetitions. In the 8-ft up-and-go test, the participant starts in a sitting position and, at the evaluator’s signal, has to get up, walk 2.44 m and then go back and sit down. The test is performed twice and counts the best result. Finally, in the 6-min walk test, the aim is to walk the longest distance over 6 min.

### 2.3. Data Analysis

The assumption of normality regarding the distribution of the data of each variable was evaluated using the Kolmogorov-Smirnov test. A descriptive analysis of main characteristics, physical function and PhA of the participants was carried out. For the comparison of variables between women and men, we used the *t*-test for independent samples for scale variables and chi-square for categorical variables. Pearson’s correlation was used to analyze the intensity and direction of the relationship between PhA and the physical fitness variables. Correlation thresholds of 0.10 were small, 0.30 were moderate and were 0.50 high [23]. Multiple linear regression was computed to examine three models: Model 1, formed by a dependent variable “phase angle” and two predictor variables, “8 ft up-and-go” and “6 min walk”; Model 2, formed by a dependent variable “phase angle” and three predictor variables, “8 ft up-and-go”, “6 min walk” and “30-s chair stand”; and Model 3, formed by a dependent variable “phase angle” and four predictor variables, “8 ft up-and-go”, “6 min walk”, “30-s chair stand” and “arm curl”. Statistical analyses were performed using the statistical package SPSS v.26 (IBM, New York, NY, USA). For all tests, the significance level was set at *p* < 0.050.

## 3. Results

Table 1 presents the demographic characteristics, showing significant differences between women and men in height, BMI, waist circumference and education level. Of the 111 participants in this study, 75 were women and 36 were men, with an average age of around 85 years old. The sample constituted volunteers of both genders, with more women. Both genders also had a similar mean age, but men had a significantly higher height. Weight was also superior for men, although showed no significant differences. BMI and waist circumference were both statistically significantly lower for men.

Results for the comparison between both genders are presented in Table 2. Only significant differences were found between women and men in meters walked in 6 min.

Table 3 shows the correlations between PhA and physical fuction. PhA significantly correlated with all physical fitness tests (*p* < 0.010 for total sample and men; and *p* < 0.050 for women), except for the arm curl test. However, these correlations were low for the total sample and for women, and medium for men.

Table 4 shows multiple linear regression was used to verify whether the independent variables described were capable of predicting the dependent variable PhA in the total sample. Model 1: F (7.686) = 10.853; *p* = 0.001; R^2^ = 0.125; Model 2: F (5.280) = 11.231; *p* = 0.002; R^2^ = 0.129; and Model 3: F (4.000) = 11.422; *p* = 0.005; R^2^ = 0.131. Models 1, 2 and 3 showed that predictor variables have a significant influence on the PhA, 12.5%, 12.9% and 13.1%, respectively.

The results of the multiple linear regression for women are shown in Table 5, as Model 1: F (3.700) = 6.076; *p* = 0.030; R^2^ = 0.093; Model 2: F (2.068) = 6.204; *p*= 0.067; R^2^ = 0.095; and Model 3: F (1.996) = 6.675; *p* = 0.105; R^2^ = 0.102. Only Model 1 showed a significant influence of predictor variables on the PhA, 9.3%.

Finally, Table 6 shows the multiple linear regression results for men, as Model 1: F (7.493) = 6.796; *p* = 0.002; R^2^ = 0.312; Model 2: F (4.925) = 6.874; *p* = 0.006; R^2^ = 0.316; and Model 3: F (3.582) = 6.878; *p* = 0.016; R^2^ = 0.316. Models 1, 2 and 3 showed that predictor variables have a significant influence on the PhA, 31.2%, 31.6% and 31.6%, respectively.

## 4. Discussion

This study analyzed the relationship between PhA and physical function in older adults living or using a daycare center in a nursing home residence.

Additionally, we compared the differences between sexes (women vs. men) and performed three multiple linear regressions with the PhA as a predictor of physical function. Our results showed significant correlations between PhA and several functional fitness tests, such as a 30-s chair stand, an 8-ft up-and-go test and a 6 min walk, for the total sample, women and men. Finally, according to Model 3, PhA explained approximately 30% of the results of functional tests for men, and approximately 13% for the total sample. However, the only significant model for women was Model 1, which could explain approximately 9%. In this sense, we confirmed the hypothesis of the present study.

Firstly, the mean value of PhA for the total sample was 3.7 ± 0.9°, for women it was 3.8 ± 0.9° and for men it was 3.6 ± 0.8°. No differences were found between the sexes. Our study revealed different values to previous studies conducted in older adults. Matias et al. [17] showed a mean value of 5.5° ± 0.7° for the total sample, 5.3 ± 0.5° for women and 5.9 ± 0.7° for men, and revealed significant differences between the sexes. Additionally, Germano et al. [24] showed mean values of 5.9 ± 0.7° for men and 5.4 ± 0.7° for women, and also showed significant differences between the sexes. Another study [25], where participants had a similar age (80.5 ± 7.0), also showed higher values than our study; 5.2 ± 1.3° for men and 5.7 ± 1.0° for women. This may have been due to specific characteristics of the study sample. Our study recruited participants that were living or using a daycare center in a nursing home residence, and these participants showed a low education level, a high age average (85.1 ± 6.8 years) and came from a rural area of the interior of the country; all are indicators of a worst physical function level.

These lower values for PhA could be explained by the age of the participants. During the aging process, there is a decrease in muscle mass values due to an increase in extracellular water, accompanied by a decrease in intracellular water [26]. It is essential to perform permanent monitoring of the PhA values in institutionalized, independent older adults, or those in the process of becoming so. The measurement of PhA is quick and simple and does not cause any discomfort to this population. It is important to increase the value of PhA in this population, because higher values are related to cellular health, especially membrane integrity [26], permeability [27], inflammation [28] and physical function [17,29]. In addition, several studies have demonstrated that a lower value of PhA is related to average life expectancy [30,31,32], which is crucial for this population.

Secondly, regarding performance in physical fitness tests, our results showed lower values when compared with previous studies conducted in older adults [17,33]. According to a reference study concerning the 30-s chair stand test [34], our study demonstrated that patients had lower values regarding body strength. In another study, Matias et al. [17] showed that, in 113 healthy older adults (67 female and 46 male), participants with an age between 75 to 84 years performed 13.5 repetitions; whereby the men performed 14.8 repetitions and the women performed 13.1 repetitions. This year, Oliveira et al. [33] postulated that 46 independent older women with a mean age of 71 years performed 25 repetitions in 30 s. Regarding the 6-min walk test, our study revealed that the total sample covered 192.2 m, women covered 175.8 m and men covered 226.3 m in 6 min. As previously mentioned, for body strength, the participants included in this study also presented lower values for cardiorespiratory capacity. In contrast, Oliveira et al. [33] showed that 46 older women covered 612.8 m, and Matias et al. [17] reported that their overall sample covered 557 m. The study of Matias et al. [17] also included the arm curl test and the 8-ft up-and-go test, and showed higher values for both tests compared to the values recorded in this study. A possible explanation for the observed differences may be related to the characteristics of the study sample, as previously mentioned. Two of the studies mentioned here were also conducted in Portugal. The participants of Matias’s study were recruited from older adult community centers in the Lisbon area (center of Portugal), while the participants of Oliveira’s study [33] were recruited from a municipally-owned corporation, “Esposende2000” (Esposende, north of Portugal).

Clearly, the reduced PhA values reflect the physical condition level of the participants. This study provides new data regarding the relationship between PhA and physical function, represented by four functional fitness tests. The present study also confirms previous studies regarding the inverse relationship between the PhA with age [35]. More studies should be performed to confirm the present results, especially with residents living in Évora city, due to the sample characteristics.

Finally, it is important to note that Model 3 (Table 4) showed that the PhA is an excellent predictor of physical function, which showed a coefficient of determination of 31%. A previous study presented a coefficient of determination of 23% for the relationship between the PhA and muscle function in 191 older women with cancer [36]. Finally, Tomeleri et al. [28] observed that PhA was a significant predictor of muscle quality and physical function, where the best model showed a coefficient of determination of 30%. Naturally, we agree with Tomeleri and colleagues regarding that muscle quality plays a key biological role in PhA values. Recently, a recent systematic review with meta-analysis by Martins et al. [1] provided new guidelines to improve the PhA values in older adults. Therefore, we recommend the performance of resistance training programs with three sessions per week; six to ten exercises with twelve repetitions per set. These programs should be continuously implemented in senior living institutions.

The present study has strengths and limitations. Approximately 70% of the sample constituted women, which may have had an influence on outcomes. Secondly, due to the fact that the present study has a cross-sectional design, we could not determine the possible causal-effect relationships. Therefore, further longitudinal and experimental studies are required to evaluate the effectiveness of PhA as a marker of functional capacity. Even so, the present results represent a relevant preliminary analysis in order to determine the reference values for PhA in participants that are living in or using a daycare center in a nursing home residence in a rural area.

## 5. Conclusions

This study determined that PhA can be a predictor of body strength, aerobic capacity and agility in institutionalized, independent older adults. Due to the time necessary to conduct several tests regarding physical function, PhA can be used as a complementary tool for monitoring physical function in this population. Therefore, we recommend that institutions for institutionalized older adults should frequently evaluate PhA, to prevent loss of functionality and autonomy.

## Figures and Tables

**Table 1:** Main characteristics of the participants.

Outcomes	Total (*n* = 111) (Mean ± SD)	Women (*n* = 75) (Mean ± SD)	Men (*n* = 36) (Mean ± SD)	*p*-Value
Age (y) ^a^	85.1 ± 6.8	85.2 ± 6.9	84.8 ± 7.0	0.809
Height (m) ^a^	1.51 ± 0.1	1.48 ± 0.1	1.57 ± 0.1	0.001
Weight (kg) ^a^	64.8 ± 11.5	63.6 ± 11.8	67.2 ± 10.4	0.123
Body mass index (kg/m^2^) ^a^	28.3 ± 4.5	28.9 ± 4.7	27.0 ± 3.9	0.035
Waist circunference (cm) ^a^	100.4 ± 11.1	101.9 ± 11.2	97.3 ± 10.4	0.040
Education level ^b^				0.001
- Unfinished studies	46 (41.4)	32 (42.7)	14 (38.9)	
- Primary school	10 (9.0)	7 (9.3)	3 (8.3)	
- Secondary school	13 (11.7)	8 (10.7)	5 (13.9)	
- University degree	42 (37.8)	28 (37.3)	14 (38.9)	

^a^ Values expressed as mean ± SD, *p*-values of *t*-test. ^b^ Values expressed as n (%), *p*-values of analysis of chi-square test.

**Table 2:** Physical fitness and phase angle of the participants.

	Total (*n* = 111)	Women (*n* = 75)	Men (*n* = 36)	*p*
30-s chair stand (rep)	7.3 ± 3.7	7.1 ± 3.8	7.6 ± 3.6	0.497
Arm curl (rep)	10.0 ± 4.1	10.0 ± 4.4	10.1 ± 3.5	0.869
8 ft up-and-go (s)	16.4 ± 10.7	17.4 ± 11.7	14.3 ± 8.1	0.143
6 min walk (m)	192.2 ± 95.1	175.8 ± 81.7	226.3 ± 111.9	0.008
Phase angle (°)	3.7 ± 0.9	3.8 ± 0.9	3.6 ± 0.8	0.669

Values expressed as mean ± SD, *p*-values of *t*-test.

**Table 3:** Pearson’s correlation between phase angle and physical function for total, women and men.

	Phase Angle		
Physical Fitness Tests	Total (*n* = 111)	Women (*n* = 75)	Men (*n* = 36)
30-s chair stand (rep)	0.288 **	0.234 *	0.443 **
Arm curl (rep)	0.058	−0.020	0.305
8 ft up-and-go (s)	−0.328 **	−0.284 *	−0.535 **
6 min walk (m)	0.310 **	0.274 *	0.460 **

Values expressed as Pearson’s r; * *p* < 0.05, ** *p* < 0.01.

**Table 4:** Multiple linear regression (backward stepwise). Phase angle as a predictor for physical function. Total sample.

	*Beta*	*t*	*p* Value	R^2^
**Model 1**			0.001	
8 ft up-and-go (s)	−0.220	−1.882	0.428	0.125
6 min walk (m)	0.170	1.455	0.606	
**Model 2**			0.002	0.129
8 ft up-and-go (s)	−0.185	−1.469	0.145	
6 min walk (m)	0.137	1.091	0.278	
30-s chair stand (rep)	0.090	0.731	0.467	
**Model 3**			0.005	0.131
8 ft up-and-go (s)	−0.179	−1.407	0.162	
6 min walk (m)	0.151	1.174	0.243	
30-s chair stand (rep)	0.099	0.795	0.428	
Arm curl (rep)	−0.050	−0.517	0.606	

*p*-values of ANOVA; R^2^ of Model 1, formed by a dependent variable “phase angle” and two predictor variables, “8 ft up-and-go” and “6 min walk”. R^2^ of Model 2, formed by a dependent variable “phase angle” and three predictor variables, “8 ft up-and-go”, “6 min walk” and “30-s chair stand”. R^2^ of Model 3, formed by a dependent variable “phase angle” and four predictor variables, “8 ft up-and-go”, “6 min walk”, “30-s chair stand” and “arm curl”.

**Table 5:** Multiple linear regression (backward stepwise). Phase angle as a predictor for physical function. Only women.

	*Beta*	*t*	*p* Value	R^2^
**Model 1**			0.030	
8 ft up-and-go (s)	−0.182	−1.199	0.235	0.093
6 min walk (m)	0.152	0.988	0.321	
**Model 2**			0.067	0.095
8 ft up-and-go (s)	−0.158	−0.964	0.338	
6 min walk (m)	0.133	0.830	0.409	
30-s chair stand (rep)	0.059	0.393	0.695	
**Model 3**			0.105	0.102
8 ft up-and-go (s)	−0.143	−0.865	0.390	
6 min walk (m)	0.160	0.972	0.334	
30-s chair stand (rep)	0.069	0.455	0.650	
Arm curl (rep)	−0.089	−0.751	0.455	

*p*-values of ANOVA; R^2^ of Model 1, formed by a dependent variable “phase angle” and two predictor variables, “8 ft up-and-go” and “6 min walk”. R^2^ of Model 2, formed by a dependent variable “phase angle” and three predictor variables, “8 ft up-and-go”, “6 min walk” and “30-s chair stand”. R^2^ of Model 3, formed by a dependent variable “phase angle” and four predictor variables, “8 ft up-and-go”, “6 min walk”, “30-s chair stand” and “arm curl”.

**Table 6:** Multiple linear regression (backward stepwise). Phase angle as a predictor for physical function. Only men.

	*Beta*	*t*	*p* Value	R^2^
**Model 1**			0.002	
8 ft up-and-go (s)	−0.407	−2.194	0.035	0.312
6 min walk (m)	0.205	1.109	0.276	
**Model 2**			0.006	0.316
8 ft up-and-go (s)	−0.375	−1.854	0.073	
6 min walk (m)	0.164	0.770	0.447	
30-s chair stand (rep)	0.089	0.409	0.685	
**Model 3**			0.016	0.316
8 ft up-and-go (s)	−0.374	−1.818	0.079	
6 min walk (m)	0.160	0.729	0.471	
30-s chair stand (rep)	0.082	0.355	0.725	
Arm curl (rep)	0.018	0.098	0.923	

*p*-values of ANOVA; R^2^ of Model 1, formed by a dependent variable “phase angle” and two predictor variables, “8 ft up-and-go” and “6 min walk”. R^2^ of Model 2, formed by a dependent variable “phase angle” and three predictor variables, “8 ft up-and-go”, “6 min walk” and “30-s chair stand”. R^2^ of Model 3, formed by a dependent variable “phase angle” and four predictor variables, “8 ft up-and-go”, “6 min walk”, “30-s chair stand” and “arm curl”.

## Data Availability

All data are presented in the manuscript.
